# Supplementary material for: Additively-manufactured monocrystalline YBCO superconductor
Source: Nat Commun. 2025 Feb 24;16:1933. doi: 10.1038/s41467-025-56708-x (PMC11850711; doi:10.1038/s41467-025-56708-x)
Supplement: Supplementary file 1 — Supplementary Information [file 41467_2025_56708_MOESM1_ESM.pdf]

Supplementary Information

Additively-manufactured monocrystalline YBCO superconductor

Dingchang Zhang[1,*], Cristian Boffo[2], David C. Dunand[1,*]

1. Department of Materials Science and Engineering, Northwestern University, Evanston, IL 60208, USA

2. Fermi National Accelerator Laboratory, Batavia, IL 60510, USA

* Corresponding authors: dunand@northwestern.edu; Phone number: +1-847-491-5370
dingchangzhang2020@u.northwestern.edu; Phone number: +1-773-865-9150

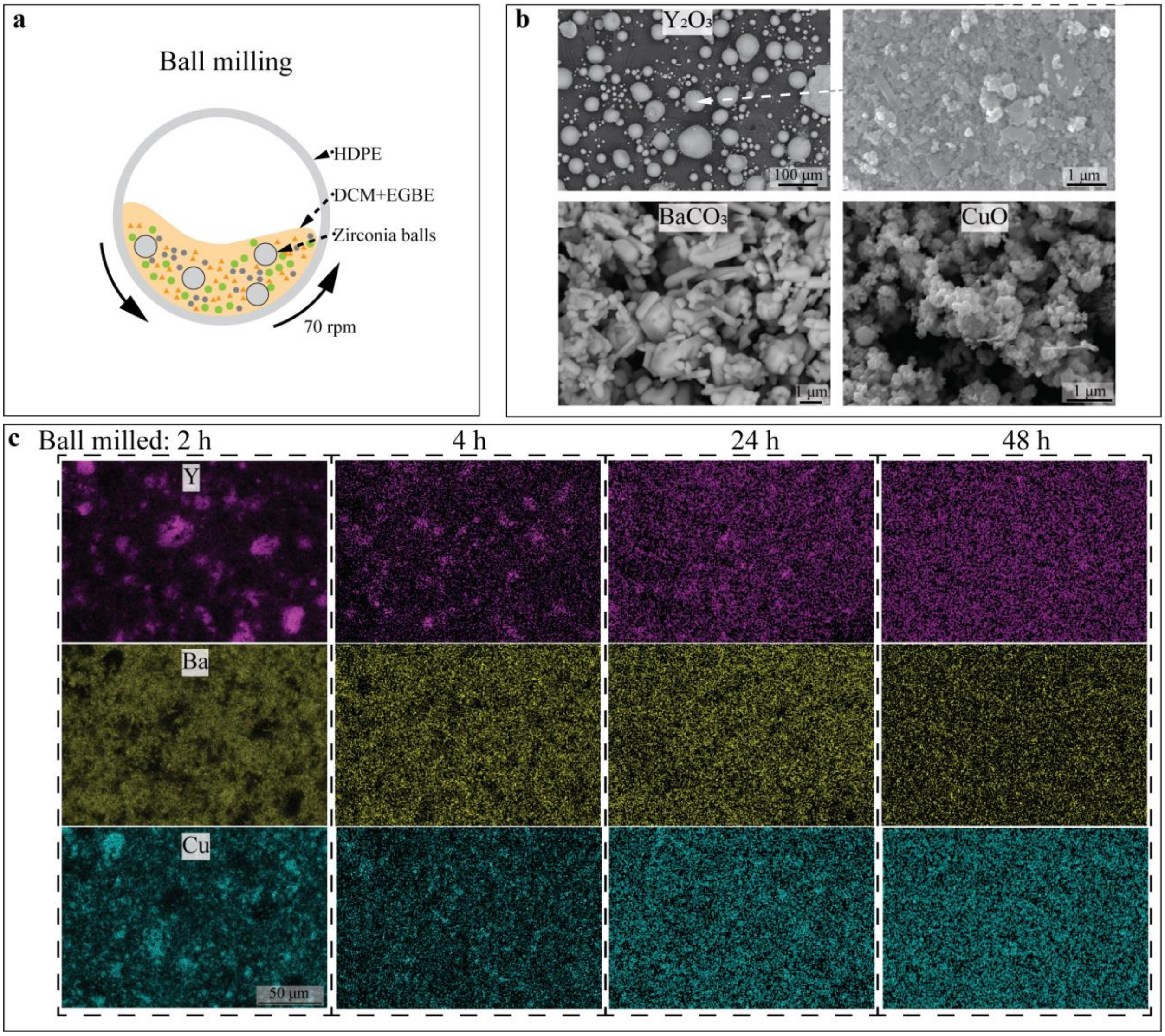

Supplementary Figure S1. Powders and mixing

(a) Schematic illustration of the ball milling process. The powder blend was roller-mixed in a High-Density Polyethylene (HDPE) bottle with zirconia balls as mixing medias. After the initial 48 hours of ball milling, PLGA and EGBE are then added for an additional 12 hours of ball milling. (b) SEM-BSE micrograph for precursor powders: Y_2O_3 (0.5-1 μm), $BaCO_3$ (0.8 μm), and CuO powder (25-55 nm). (c) Energy-Dispersive Spectroscopy (EDS) micrographs of powder blends, showing uniform distribution of Y, Ba, and Cu elements after ball milling at 2, 4, 24, and 48 h with a ball-to-powder weight ratio of 2:1. A 10 nm Y_2O_3 powder is used for these blend, which is replaced by 0.5-1 μm Y_2O_3 powders for all other samples to prevent accidental loss by electrostatic separation during handling. Powders can be de-agglomerated and mixed homogeneously after ball milling of 48 h. The ball-to-powder weight ratio is increased to 5:1 with a mixing time of 48 h for all other samples for better mixing.

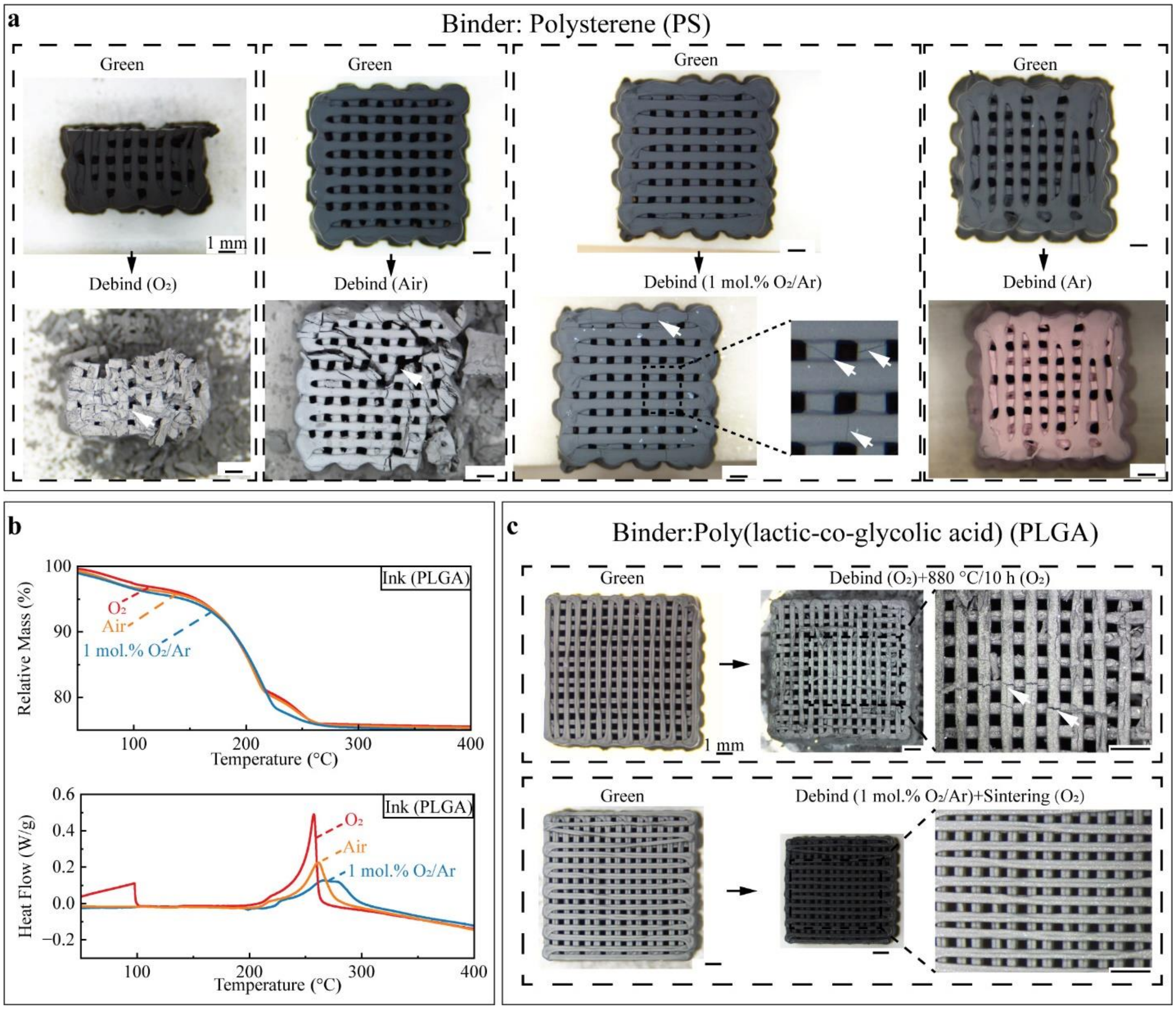

Supplementary Figure S2. Debind

(a) Photographs of a 3D printed micro-lattice before and after polystyrene debinding at 450 ˚C for 30 min under four atmospheres (pure O_2, air, Ar-1 mol.% O_2, and pure Ar). Cracks (arrows) are formed when the sample debinds under pure O_2, air, and Ar-1% O_2. The red color of the micro-lattice after Ar debinding indicates reduction of CuO by carbon-rich decomposition products of polystyrene. Coarsening of nano-Cu particles is expected to happen after reduction. (b) TGA and DSC plots of ink subjected to PLGA debinding, upon heating from 50 to 400 ˚C under pure O_2, air, and Ar-1% O_2 (50 ml/min). The exothermic heat signal decreases as the oxygen content in the gas decreases, indicating a reduction in combustion of binder (or their decomposition products) at a low oxygen partial atmosphere. (c) Top: photographs of 3D printed lattice before and after PLGA debinding (300 ˚C/ 30 min) under pure O_2 followed by sintering at 880 ˚C for 10 h under O_2; some cracks are marked with arrows. Bottom: photographs of 3D printed micro-lattice before and after PLGA debinding (300 ˚C/ 30 min) under Ar-1% O_2 and sintering at 1000 ˚C for 20 h under O_2; no cracks are found when PLGA is debinded s Ar-1% O_2.

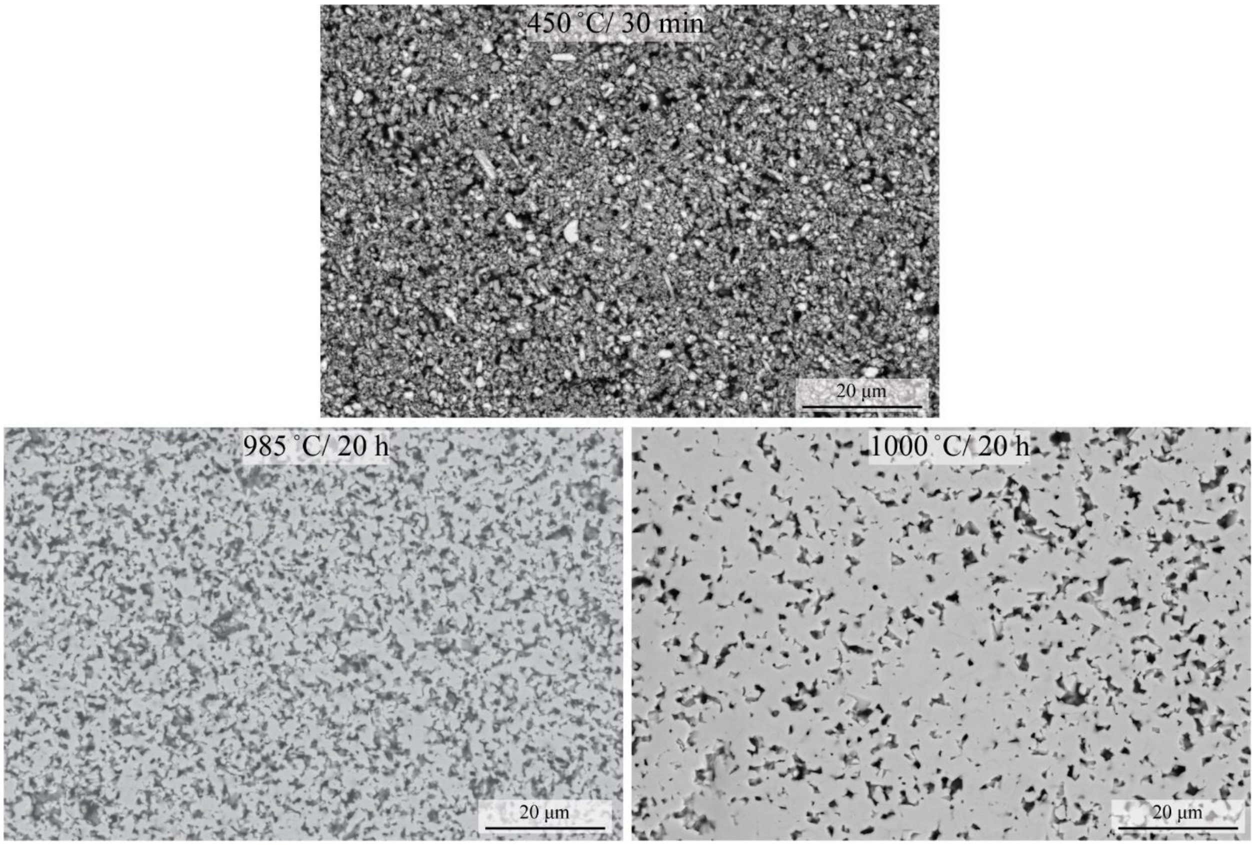

Supplementary Figure S3. Sintering utilizing polystyrene as a binder

SEM-BSE micrographs of the cross-section of 3D printed micro-lattice samples after debinding at 450 ˚C for 30 min, sintering at 985 ˚C for 20 h, and sintering at 1000 ˚C for 20 h under pure O_2. The binder used is polystyrene. The SEM figures are taken in crack-free regions. The sample with polystyrene as binder shows a similar microstructure shown in Figure 1 (f) at 1000 ˚C for 20 h.

Supplementary Information SI1 - Percolation threshold of Y_2BaCuO_5 (Y211) and liquid:

Xu et al. simulated the continuum percolation of inter-penetrable sphero-cylinders with different aspect ratios by Monte Carlo simulations[1]. They gave an empirical approximation of the percolation threshold $\emptyset_c$ from their simulation results:

$$\emptyset_c = 1 - e^{\left[-\frac{C(\alpha)}{V_{dex}}\right]} \qquad (S1)$$

$$C(\alpha) = 1 + (0.136169\alpha + 0.165568)^{-0.3235} \qquad (S2)$$

$$V_{dex} = 2 + \frac{6(1+\alpha)(1+0.5\alpha)}{1+1.5\alpha} \qquad (S3)$$

where α=L/D.

Priour et al. simulated the percolation through voids around cylindrical particles [2]. The percolation threshold of voids (or liquid) around the cylindrical particles with an aspect ratio of α=3 is 4 %. Therefore, a gray dotted line representing this percolation threshold for the liquid phase is shown in the Y_2O_3–BaO–CuO phase diagram, Figure S4 (c).

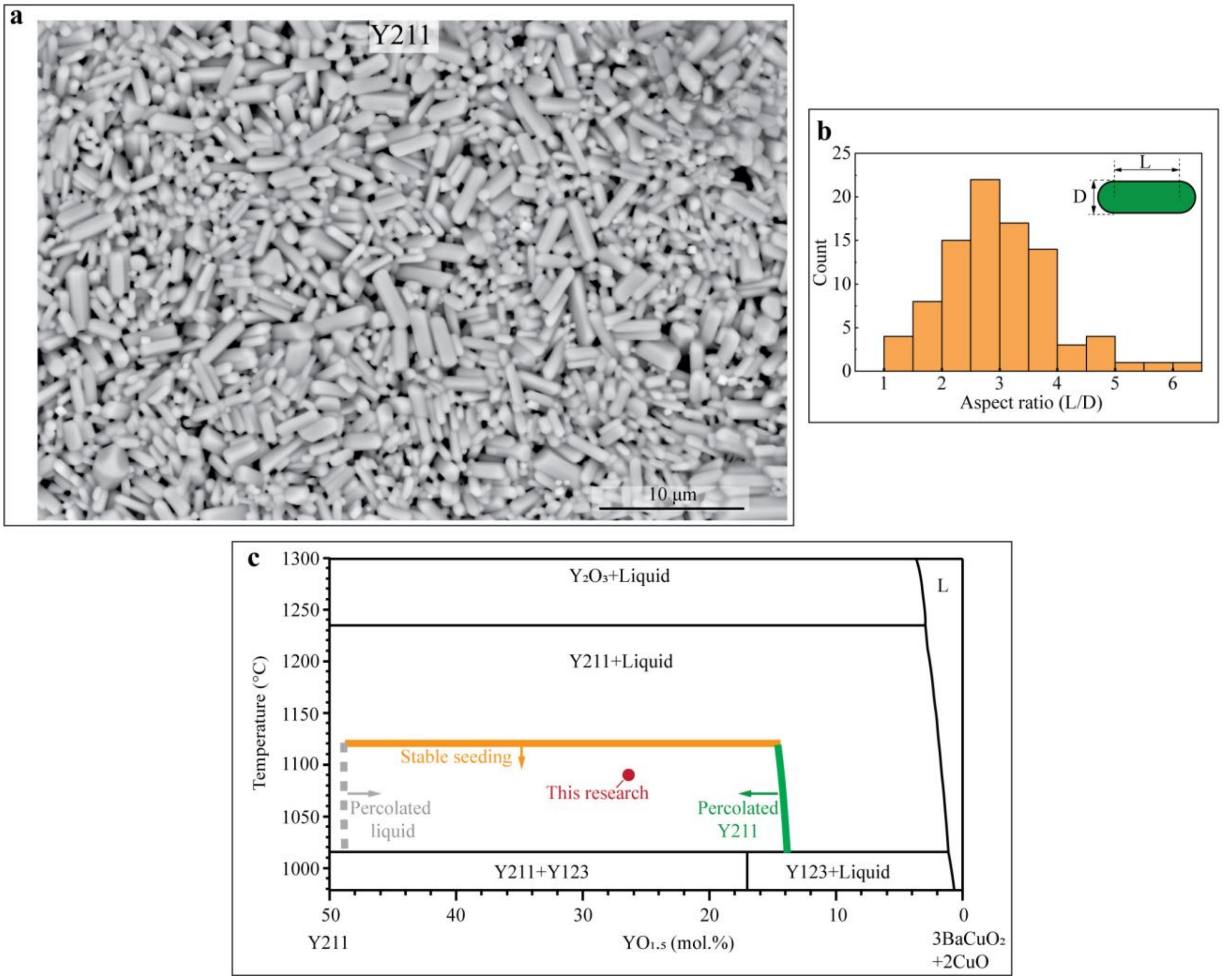

Supplementary Figure S4. Percolation threshold

(a) SEM-BSE micrograph of the Y211 particles, imaged on the liquid-depleted surface (green color region) of 3D printed micro-lattice sample after single crystal growth. (b) The distribution for the aspect ratios of Y211 particles measured from (a). The Y211 particles whose main axis is close to being in-plane are selected for measurements. (c) The vertical section of Y_2O_3–BaO–CuO phase diagram in air[3]. The liquidus line is from experimental data[4]. The percolation thresholds for Y211 particles (green line) and liquid phase (gray line) are shown. The maximum temperature (~1120 ˚C) that NdBCO seeds can be used is shown as orange line[5,6]. The composition of current research is shown as a red dot and corresponds to 51 mol.% (or 36 vol.%) of solid Y211 particles. Here, the molar volume of the liquid phase is assumed to be the same as that of $3BaCuO_2$+2CuO (“$Ba_3Cu_5O_8$”) for calculating mole fraction from volume fraction.

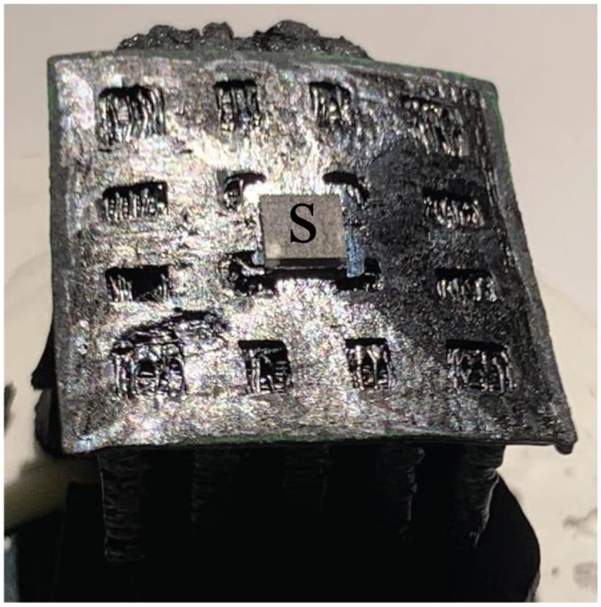

Supplementary Figure S5. Fan-shaped growth surface

A photograph of another horizontal coil sample with a clearly visible fan-shaped growth surface, showing four distinct growth sectors after single crystal growth. A mesh-like surface was used to reduce friction between the sample and the substrate during sintering. The square surface of seed (labeled as “S”) has a dimension of 2×2 mm^2.

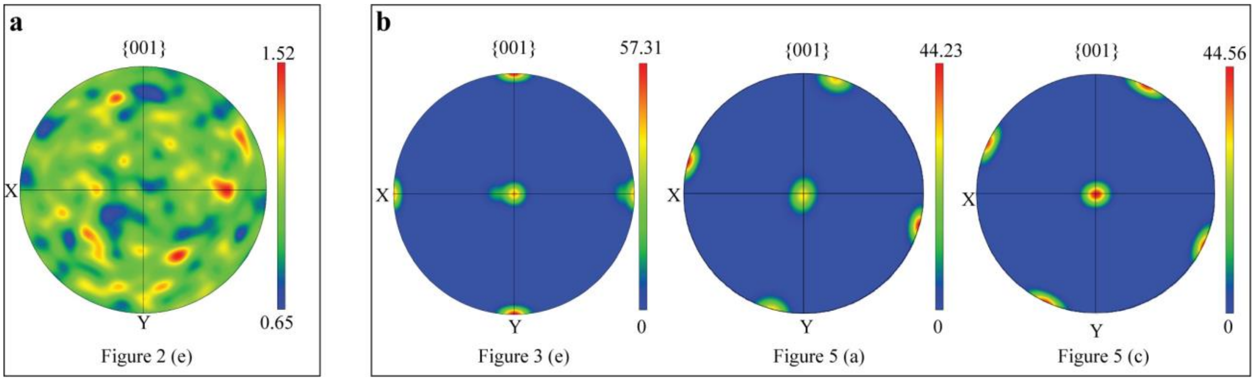

Supplementary Figure S6. Pole Figures

Pole figures for polycrystal microstructures shown in Figure 2 (e), and single-crystal microstructures shown in Figure 3 (e), in Figure 5 (a) - top view of the sample, and in Figure 5 (c).

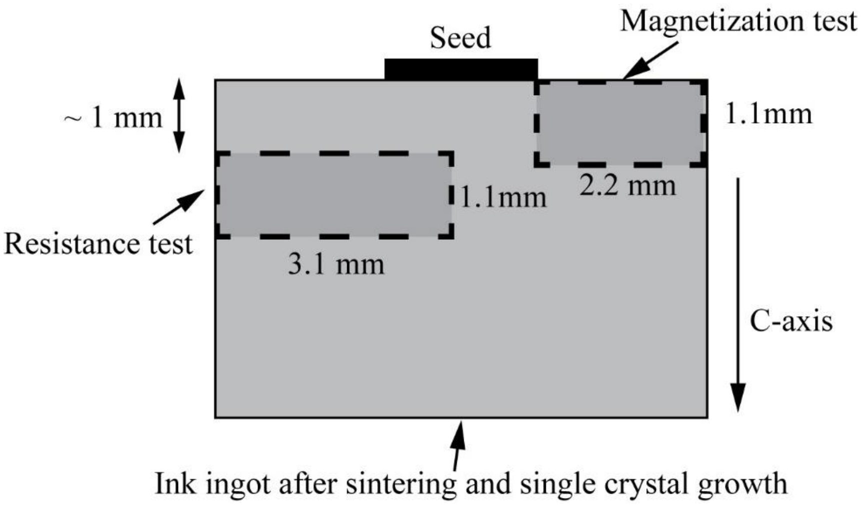

Supplementary Figure S7. Samples' location and size

Schematic figure showing an ink ingot after sintering and single-crystal growth. The locations and dimensions of the two samples used for magnetization ($2.2 \times 1.1 \times 1.8$ mm^3) and resistance measurements (half cylinder, a radius of 3.1 mm and a thickness of 1.1 mm) are labeled.

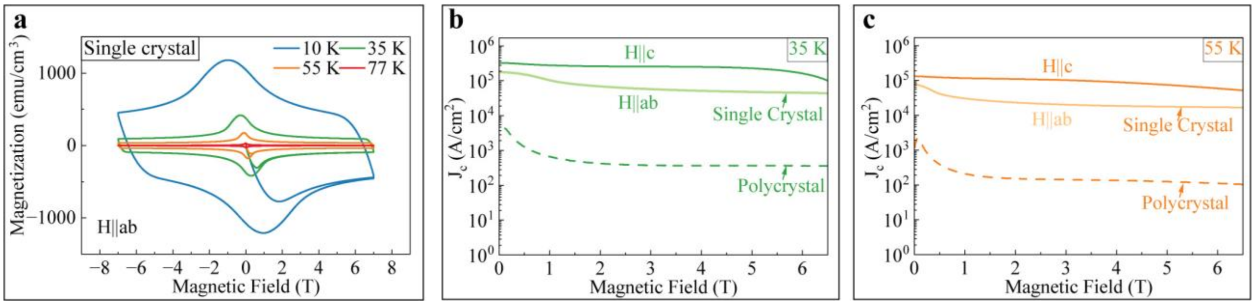

Supplementary Figure S8. Magnetization tests

(a) Plot of magnetization vs. magnetic field for single-crystal samples when the magnetic field is parallel to the a/b axis of Y123. (b,c) Critical current density as a function of magnetic field for poly- and monocrystalline (H||c and H||ab) YBCO at (b) 35 K and (c) 55 K.

Supplementary Information SI2 – Effect of impurity elements:

Supplementary Table S1. Impurity element concentrations (as measured from ICP-AES) for Y123-Y211 samples sintered at 1000 ˚C for 20 h.

Element	As	B	Ca	Co	Fe	K	Na	Si	Ti
wt. %	0.02	0.014	0.09	0.01	0.01	0.01	0.06	0.11	0.03

*: other elements tested (<0.01 wt.%): Al, Be, Bi, Cd, Cr, Dy, Er, Eu, Ga, Ge, Gd, Hf, Ho, In, La, Li, Lu, Mg, Mn, Mo, Nb, Nd, Ni, P, Pb, Pr, Rb, Re, Sb, Sc, Se, Sm, Sn, Sr, Ta, Tb, Te, Th, Tl, Tm, U, V, W, Yb, Zn, Zr

Nine impurity elements, shown in Table S1, are present above the background level of 0.01 wt.%: As, B, Ca, Co, Fe, K, Na, Si, and Ti. Most of these impurities have a negative effect on the superconducting properties of Y123, as described below. These impurities (summing to 0.35 wt.%) are thus, very likely, the reason for a depressed T_c value of 88-89.5 K and a relatively low J_c (2.1×10^4 A·cm^{-2}, H||c), as shown in Figure 3, compared with traditional Y123+Y211 fabricated by top-seeded melt growth.

1. Arsenic. A Y123 film grown on a MgO/GaAs substrate showed a relatively low T_c (89 K) [7], with a relatively low critical current density J_c ($4\text{-}6.7 \times 10^4$ A·cm^{-2} at 77K) compared with the Y123 grown on pure MgO (10^6 A·cm^{-2} at 77K)[7]. This was attributed to the contamination by As diffused or evaporated from the MgO/GaAs substrate [7].
2. Boron. The effect of B has been studied by adding B_2O_3 powders to a Y123 sample [8]. The addition of 0.05 wt.% of B_2O_3 (B: 0.016 wt.%) decreases T_c by ~ 1.6 K and decreases the critical current density from 1.6 to 1.2×10^4 A·cm^{-2} at 50 K under self-field [8]. The B content in our sample is 0.014 wt.%, similar to the above value.
3. Calcium. Addition of Ca into a Y123 thin film was detrimental to T_c and J_c [9] by cation substitution of Y^{3+} with Ca^{2+} and formation of oxygen vacancies. A ~0.30 wt. % Ca concentration decreases T_c to 80-85 K and depresses J_c by four orders of magnitude, from ~10^6 A·cm^{-2} (Ca free) to ~10^5 A·cm^{-2}at 77 K under self-field [9]. The Ca content (0.09 wt.%) in our sample is one third of the above value of 0.30 wt.%, but still very likely to lower T_c and J_c.
4. Cobalt. Addition of ~0.18 wt.% Co in Y123 decrease its T_c by ~1 K[10].
5. Iron. Addition of ~ 0.02 wt.% Fe in Y123 leads a T_c of 87-90 K[11]. Our Fe content (0.01 wt.%) is half the above value
6. Potassium. Addition of ~0.01 wt.% K in Y123 decreased T_c by ~0.5 K[12]. We have the same K content (0.01 wt.%).
7. Sodium: Y123 with 0.24 wt.% Na shows a limited effect on the onset of superconducting transition temperature T_c[13]. The value of J_c is also not apparently depressed by 0.24 wt.% Na in Y123[13]. Therefore, 0.06 wt.% Na in our samples is not expected to affect superconducting properties.
8. Silicon. The addition of 0.1 to 0.5 wt.% SiO_2 (Si: 0.05 to 0.23 wt.%) has a limited effect on T_c[14]. Also, the addition of 0.09 wt.% Si did not cause an apparent drop in critical current densities under a self-field[14].

9. Titanium. Y123 with 0.36 wt.% Ti shows a strongly reduced value of T_c of 76 K [15]. Our sample has a much lower Ti content (0.03 wt.%) but Ti may still contribute to a lower T_c.

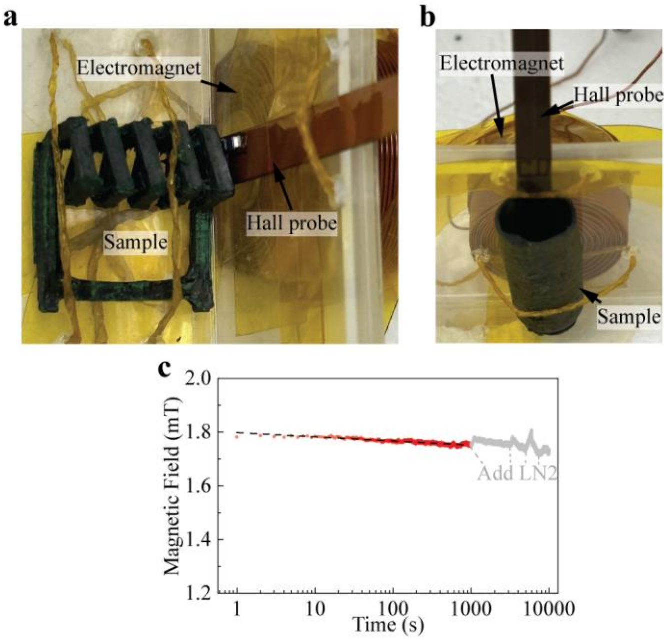

Supplementary Figure S9. Experimental setup and data

Photographs of setup to test the (a) coil loop and (b) hollow tube (height: 18.1 mm, mean outer radius : 8.6 mm, wall thickness : 0.6 mm, and mean inner radius: 8.0 mm). (c) The evolution of the generated magnetic field of the coil loop as a function of time was extended up to 10,000 seconds. The additional noise beyond 1000 sec is due to small shifts of the probe from adding LN_2.

Supplementary Information SI3 - Current and flux creep in the coil loop:

Here, we assume that the measured magnetic field comes from a persistent current along the path of the horizontal coil rather than from microscopic current vortices. Each turn of the coil is simplified as a square loop. The magnetitic field $B(x)$ generated from a square loop along its normal axis is given by [16]:

$$B(x) = \frac{\mu_0 I}{4\pi} l^2 \left(x^2 + \frac{l^2}{4}\right)^{-1} \left(x^2 + \frac{l^2}{2}\right)^{-\frac{1}{2}} \qquad (S4)$$

where μ_0 is the vacuum magnetic permeability, I is current, l is the width of the square loop, and x is the distance to the center of the square loop. The width for each square loop is 4 mm. The distance between each turn of the coil and Hall probe are 11.6, 8.7, 5.9, 2.6, and 0 mm. The initial

measured magnetic field is 1.8 mT. Therefore, the current in the coil is calculated as 9.5 A or ~560 A·cm^{-2}. A higher persistent current in the coil can be achieved by using flux pumping [17].

The logarithmic decay of the measured magnetic field is due to the magnetic flux creep, which can be fitted with[18]:

$$B(x) = a - bln\frac{t}{t_0} \qquad (S5)$$

where t is time and t_0 is the unit of time. Fitting of data shown in Figure 4 (a) before adding LN2 during measurement (1000 s) provides the following parameters: a = 18.0 G, b = 0.067 G. Therefore, the time to lose 1 % of the original value is 15 s and the time to lose 5 % of the original value is 189 h. Rong et al.[19] studied the logarithmic decay of magnetic field for YBCO closed loops made by cutting middle slits on YBCO coated tapes. They found that the time to lose 1 % of the original value was 126 s. A higher critical current density (stronger flux pinning force) of these YBCO-coated tapes is the possible reason for their lower decaying rate.

Supplementary References

[1] Xu W, Su X, Jiao Y. Continuum percolation of congruent overlapping spherocylinders. Physical Review E 94, 032122 (2016).

[2] Priour DJ, McGuigan NJ. Percolation through Voids around Randomly Oriented Polyhedra and Axially Symmetric Grains. Phys Rev Lett 121, 225701 (2018).

[3] Krabbes G, Fuchs G, Canders W-R, May H, Palka R. High temperature superconductor bulk materials. NJ, Hoboken: Wiley-VCh Darmstadt, (2006)

[4] Krauns C, Sumida M, Tagami M, Yamada Y, Shiohara Y. Solubility of RE elements into Ba−Cu−O melts and the enthalpy of dissolution. Zeitschrift für Physik B Condensed Matter 96, 207-212 (1994).

[5] Volochová D, Kavečanský V, Antal V, Diko P, Yao X. Thermal stability of NdBCO/YBCO/MgO thin film seeds. Supercond Sci Technol 29, 044004 (2016).

[6] Xiang H, et al. Film thermal stability correlation with seeding modes in the growth of YBa2Cu3O7-δ crystals. J Appl Crystallogr 49, 873-879 (2016).

[7] L.D. Chang, M.Z. Tseng, E.L. Hu, D.K. Fork, Epitaxial MgO buffer layers for $YBa_2Cu_3O_{7-x}$ thin film on GaAs, Appl. Phys. Lett. 60(14) (1992) 1753-1755.

[8] U. Topal, H. Ozkan, Effects of B_2O_3 addition on the properties of melt-processed $YBa_2Cu_3O_{7-\delta}$, Supercond. Sci. Technol. 18(1) (2005) 82.

[9] A. Augieri, T. Petrisor, G. Celentano, L. Ciontea, V. Galluzzi, U. Gambardella, A. Mancini, A. Rufoloni, Effect of Ca doping in YBCO superconducting thin films, Physica C: Superconductivity 401(1) (2004) 320-324.

[10] L. Liu, C. Dong, J. Zhang, J. Li, The microstructure study of Co-doped YBCO system, Physica C: Superconductivity 377(3) (2002) 348-356.

[11] X. Yao, A. Oka, T. Izumi, Y. Shiohara, Crystal growth and superconductivity of Fe-doped YBCO single crystals, Physica C: Superconductivity 339(2) (2000) 99-105.

[12] C.S. Tan, M.M. Awang Kechik, N.A. Che Dzulkifli, S.I. Sukor, A.N. Kamarudin, S.H. Yap, H. Baqiah, M.K.A. Karim, S.K. Chen, K.P. Lim, K.K. Mohd Shariff, S.A. Halim, Effect of concentration of potassium added in Y1Ba2Cu3O7-δ superconductor by using the thermal treatment method, AIP Conference Proceedings 2619(1) (2023).

[13] X.W. Zou, Z.H. Wang, H. Zhang, The peak effect observed in the Na substituted melt textured growth YBCO, Physica C: Superconductivity 356(1) (2001) 39-45.

[14] M.K. Ben Salem, E. Hannachi, Y. Slimani, A. Hamrita, M. Zouaoui, L. Bessais, M. Ben Salem, F. Ben Azzouz, SiO2 nanoparticles addition effect on microstructure and pinning properties in YBa2Cu3Oy, Ceram. Int. 40(3) (2014) 4953-4962.

[15] M. Sahoo, D. Behera, Effect of Ti Doping on Structural and Superconducting Property of YBa2Cu3O7−yHigh TcSuperconductor, Journal of Superconductivity and Novel Magnetism 27(1) (2014) 83-93.

[16] Garretson, Craig M. "A combined form of the laws of Ampere and Biot–Savart." *American Journal of Physics* 54.3 (1986): 253-258.

[17] C. Hoffmann, D. Pooke and A. D. Caplin, "Flux Pump for HTS Magnets," in *IEEE Transactions on Applied Superconductivity*, vol. 21, no. 3, pp. 1628-1631, June 2011, doi: 10.1109/TASC.2010.2093115.

[18] Anderson, Philip W., and Y. B. Kim. "Hard superconductivity: theory of the motion of Abrikosov flux lines." *Reviews of modern physics* 36.1 (1964): 39.

[19] Rong CC, Barnes PN, Levin GA, Miller JD, Santosusso DJ, Fitzpatrick BK. Investigation of the Relaxation of Persistent Current in Superconducting Closed Loops Made Out of YBCO Coated Conductors. *IEEE Transactions on Applied Superconductivity* **25**, 1-5 (2015).
