# Supplementary material for: Additively-manufactured monocrystalline YBCO superconductor
Source: Nat Commun. 2025 Feb 24;16:1933. doi: 10.1038/s41467-025-56708-x (PMC11850711; doi:10.1038/s41467-025-56708-x)
Supplement: Supplementary file 2 — Description of Additional Supplementary Files [file 41467_2025_56708_MOESM2_ESM.pdf]

Description of Additional Supplementary Files

Supplementary Movie 1

Title: Additive Manufacturing of the Coil Loop

Description: The additive manufacturing process of a larger Coil Loop with 7 turns showing the successful printing of overhanging bridges.

Supplementary Movie 2

Title: Levitation of the Coil Loop

Description: The levitation of the additively-manufactured monocrystalline Coil Loop on four Nd-Fe-B magnets after immersing the item into liquid nitrogen.

Supplementary Movie 3

Title: Levitation of the Cylinder

Description: The levitation of the additively-manufactured monocrystalline Cylinder on four Nd-Fe-B magnets after immersing the item into liquid nitrogen.

Supplementary Movie 4

Title: Levitation of the Toroidal Coil

Description: The levitation of the additively-manufactured monocrystalline Toroidal Coil on four Nd-Fe-B magnets after immersing the item into liquid nitrogen.

Supplementary Movie 5

Title: Origami Folding of the Boat

Description: The Origami folding process of an additively-manufactured rectangular plate, obtaining a boat-shaped item.

Supplementary Movie 6

Title: Levitation of the Origami Items

Description: The levitation of the Origami items, including the polycrystalline boat, plane, and twisted lattice band, on four Nd-Fe-B magnets after immersing items into liquid nitrogen.
